# Distinct Exosomal miRNA Profiles from BALF and Lung Tissue of COPD and IPF Patients

**DOI:** 10.3390/ijms222111830

**Published:** 2021-10-31

**Authors:** Gagandeep Kaur, Krishna Prahlad Maremanda, Michael Campos, Hitendra S. Chand, Feng Li, Nikhil Hirani, M. A. Haseeb, Dongmei Li, Irfan Rahman

**Affiliations:** 1 Department of Environmental Medicine, University of Rochester Medical Center, Rochester, NY 14642, USA; Gagandeep_Kaur@URMC.Rochester.edu (G.K.); or kmaremanda@bwh.harvard.edu (K.P.M.); 2 Division of Pulmonary, Allergy, Critical Care, University of Miami School of Medicine, Miami, FL 33136, USA; mcampos1@med.miami.edu; 3 Department of Immunology and Nanomedicine, Florida International University, Miami, FL 33199, USA; hchand@fiu.edu; 4 Centre for Inflammation Research, Queen’s Medical Research Institute, University of Edinburgh, Edinburgh EH16 4TJ, UK; Feng.Li@ed.ac.uk (F.L.); N.Hirani@ed.ac.uk (N.H.); 5 Department of Cell Biology, SUNY Downstate Health Sciences University, Brooklyn, NY 11203, USA; Haseeb.Siddiqi@downstate.edu; 6 Clinical and Translational Science Institute (CTSI), Public Health Sciences, University of Rochester Medical Center, Rochester, NY 14642, USA; Dongmei_li@urmc.rochester.edu

**Keywords:** exosomes, miRNA, COPD, biomarker, BALF, lungs

## Abstract

Chronic obstructive pulmonary disease (COPD) and idiopathic pulmonary fibrosis (IPF) are chronic, progressive lung ailments that are characterized by distinct pathologies. Early detection biomarkers and disease mechanisms for these debilitating diseases are lacking. Extracellular vesicles (EVs), including exosomes, are small, lipid-bound vesicles attributed to carry proteins, lipids, and RNA molecules to facilitate cell-to-cell communication under normal and diseased conditions. Exosomal miRNAs have been studied in relation to many diseases. However, there is little to no knowledge regarding the miRNA population of bronchoalveolar lavage fluid (BALF) or the lung-tissue-derived exosomes in COPD and IPF. Here, we determined and compared the miRNA profiles of BALF- and lung-tissue-derived exosomes of healthy non-smokers, smokers, and patients with COPD or IPF in independent cohorts. Results: Exosome characterization using NanoSight particle tracking and TEM demonstrated that the BALF-derived exosomes were ~89.85 nm in size with a yield of ~2.95 × 10^10^ particles/mL in concentration. Lung-derived exosomes were larger in size (~146.04 nm) with a higher yield of ~2.38 × 10^11^ particles/mL. NGS results identified three differentially expressed miRNAs in the BALF, while there was one in the lung-derived exosomes from COPD patients as compared to healthy non-smokers. Of these, miR-122-5p was three- or five-fold downregulated among the lung-tissue-derived exosomes of COPD patients as compared to healthy non-smokers and smokers, respectively. Interestingly, there were a large number (55) of differentially expressed miRNAs in the lung-tissue-derived exosomes of IPF patients compared to non-smoking controls. Conclusions: Overall, we identified lung-specific miRNAs associated with chronic lung diseases that can serve as potential biomarkers or therapeutic targets.

## 1. Introduction

Tobacco smoking remains the most prevalent preventable cause of morbidity and mortality worldwide. Comprising of more than 5000 compounds [1], cigarette smoke is the leading risk factor for developing chronic obstructive pulmonary disease (COPD) and idiopathic pulmonary fibrosis (IPF) in humans. Despite their distinct clinical features, both COPD and IPF are defined as severe, progressive airway diseases associated with increased risk of cancer development [2,3]. While COPD is characterized by an irreversible and progressive airflow limitation due to emphysema and bronchitis, IPF is characterized by thickening and scarring of lung tissue with fibrotic foci, thus leading to impaired diffusion capacity [3]. The current therapies for these conditions are mainly palliative, and the chief reason of this is due to limited understanding of the pathophysiology of the respective ailment [4,5].

Evidence from literature suggests the role of extracellular vesicles (EVs), specifically exosomes, in the disease severity and outcome in COPD and IPF [6,7,8,9,10]. Exosomes are a subtype of EVs formed by an endosomal route with a diameter of 30–150 nm [11]. They function to maintain the tissue homeostasis and intracellular stability [12]. However, they also become pathosomes due to harmful stimuli (e.g., tobacco smoke) and can participate in the progression of diseases. In this respect, EVs/exosomes can cause pathological changes, including oxidative stress, chronic inflammation, apoptosis, aging, epigenetic alterations, and multi-organ dysfunction in COPD [13,14,15,16]. Interestingly, exosomes are produced and released in large numbers in the sputum, serum, and BALF of COPD patients, which makes them a useful target to develop non-invasive diagnostics in COPD. Similarly, exosomes isolated from the biological fluids cause pro-inflammatory responses in lung cells [13,17,18]. Previous studies have mostly compared the serum-derived exosome populations from COPD patients and healthy individuals [19,20,21,22,23,24]. In fact, we have also compared the miRNA profiles of plasma-derived EVs from COPD patients versus healthy smokers and non-smokers [13]. However, there is little to no knowledge about the BALF or the lung-tissue-derived exosome populations in COPD or IPF.

Furthermore, numerous studies have shown that circulating miRNAs are involved in the progression, development, and severity of various diseases, including COPD and IPF [6,9,13,17,18,25,26]. Several circulating miRNAs are also being considered as plausible targets for biomarker development [27,28]. Based on this, we compared the miRNA population in the BALF and lung-tissue-derived exosomes (i.e., exosome-enriched EVs) from healthy non-smokers (NS), healthy smokers (Sm), and patients with COPD and IPF in several independent cohorts to identify potential biomarkers to determine the extent of any pulmonary damage at an early stage.

## 2. Results

### 2.1. Characterization of BALF and Lung-Tissue-Derived Exosomes

**Figure 1:**
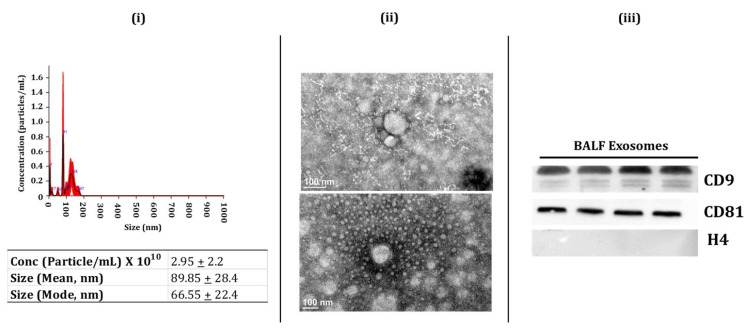
Characterization of human BALF-derived Exosomes (i.e., exosome-enriched EVs). (**i**) Representative image for particle size distribution of BALF-derived exosome in one sample as estimated using NanoSight NS300. Average particle size depicted as mean, mode, and particle concentration in BALF-derived exosome samples (*n* = 3–8/group). (**ii**) Representative TEM images of BALF-derived exosomes (*n* = 3). (**iii**) Immunoblot analysis of positive (CD9 and CD81) and negative (H4) exosomal markers derived from human BALF (*n* = 4).

**Figure 2:**
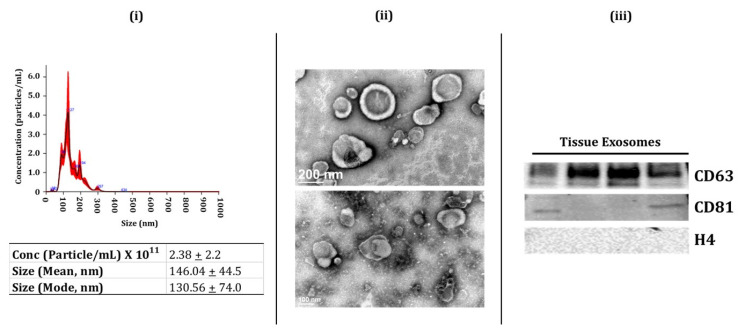
Characterization of human lung-tissue-derived Exosomes (i.e., exosome-enriched EVs. (**i**) Representative image for particle size distribution of lung-tissue-derived exosome in one sample as estimated using NanoSight NS300. Average particle size depicted as mean, mode, and particle concentration in lung-tissue-derived exosome samples (*n* = 3–5/group). (**ii**) Representative TEM images of lung-tissue-derived exosomes (*n* = 6). (**iii**) Immunoblot analysis of positive (CD63 and CD81) and negative (H4) exosomal markers derived from human lung tissue (*n* = 4).

Exosomes are involved with intercellular communication, thus affecting the physiological processes in various tissues [11,29]. Here, we analyzed the miRNA population from the BALF and lung tissue-derived exosomes isolated from non-smokers, smokers, and patients with COPD or IPF. We first isolated the BALF and lung-tissue-derived exosomes using the methods described below. We employed immunoblotting, nanoparticle tracking analysis (NTA: NanoSight 300), and transmission electron microscopy (TEM) to characterize the isolated exosomes per the regulations of ISEV [30]. We first used NTA to determine the particle concentration, size, or distribution of exosomes in isolated samples from BALF and lung tissues. The lung-derived exosomes (avg. conc. = 2.38 ± 2.2 × 10^11^ particles/mL) had a larger size (mean = 146.04 nm). On the other hand, the average size of the BALF-derived exosomes was ~89.85 nm (avg. conc. = 2.95 ± 2.2 × 10^10^ particles/mL) (Figure 1i and Figure 2i). TEM analysis confirmed the morphology of isolated exosomes from BALF and lung tissue samples, as shown in Figure 1ii and Figure 2ii. It is important to mention here that we did not observe a significant change in the exosomes isolated from the BALF and lung tissues from various sub-groups (NS, Sm, COPD, and IPF).

Finally, we used immunoblotting to study the presence of exosome surface markers (CD9, CD81, and CD63) in the isolated exosome fractions from the BALF and lung tissues. We found enrichment of positive surface markers for BALF exosomes, such as CD9 and CD81, in the isolated exosome fractions (Figure 1iii, full blots in Supplementary Figure S1). Similarly, we found an abundance of positive surface markers for tissue exosomes- CD63 and CD81- in the exosome fractions from the lung tissue (Figure 2iii, full blots in Supplementary Figure S2). We also probed for histone 4 (H4) for both BALF- and lung-tissue-derived exosomes as a negative marker and found no bands for this marker in our isolated exosomal fractions (Figure 1iii and Figure 2iii, full blots in Supplementary Figure S3). Overall, our results confirm the successful isolation of exosomes from human BALF and lung tissue in our study groups.

### 2.2. Batch Variations in the Exosome-Derived miRNA Expression Profiles amongst the Various Study Groups

**Figure 3:**
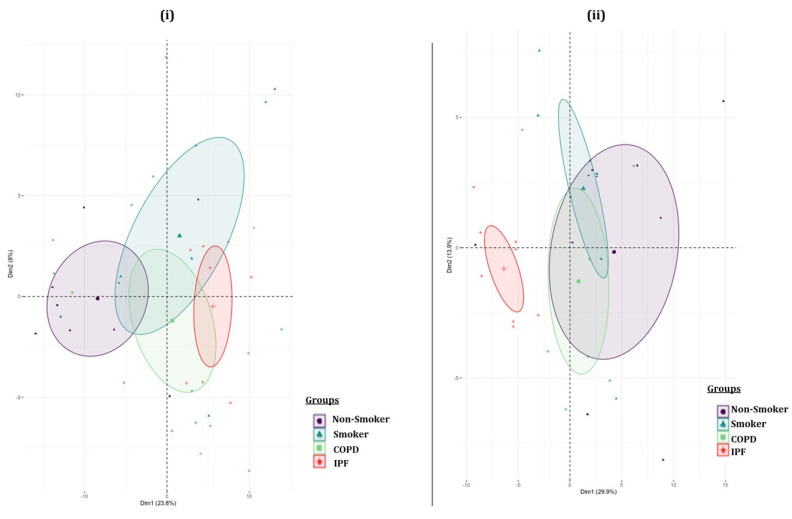
Principal component plot. Principal component analyses based on differential microRNA expression in individual (**i**) BALF- and (**ii**) lung-tissue-derived exosome samples from non-smokers, cigarette smokers, and COPD and IPF subjects.

We performed principal component analyses (PCA) to visualize the batch variations within the samples. Separate analyses were run for the BALF- and lung-tissue-derived exosomes. The plot was generated by using 50 miRNAs with the highest component of variation among groups. Each sample group was clustered using a confidence ellipse, as shown in the Figure 3. The PCA plot from lung-derived exosomal miRNAs showed a distinct clustering of the IPF patient samples as compared to the other three study groups, thus suggesting a unique transcriptomic identity of these lung-derived exosomes. 

### 2.3. Pairwise Comparison of BALF- and Lung-Tissue-Derived Exosomal miRNA Expression Profiles

**Figure 4:**
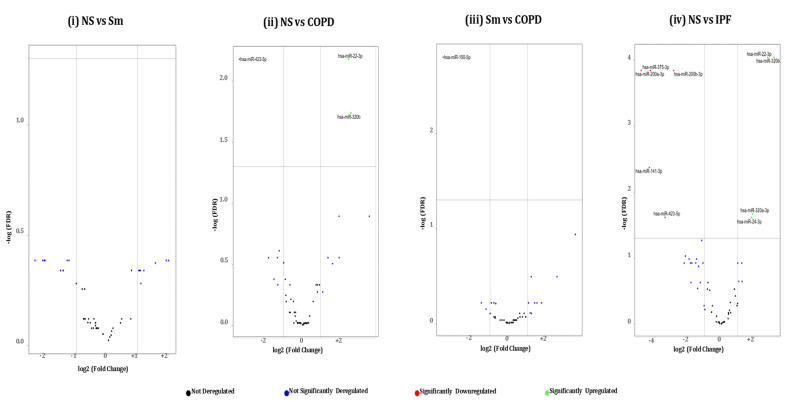
Volcano plots showing number and distribution of miRNAs from BALF-derived exosomes. Volcano plots showing the relation between −log(FDR) (y-axis) vs. log2 (fold change) (x-axis) in the differentially expressed miRNAs amongst BALF exosomes derived from (**i**) healthy non-smokers (NS) vs. healthy cigarette smokers (Sm), (**ii**) healthy non-smokers (NS) vs. COPD patients (COPD), (**iii**) healthy cigarette smokers (Sm) vs. COPD patients, and (**iv**) healthy non-smokers (NS) and IPF patients (IPF).

**Figure 5:**
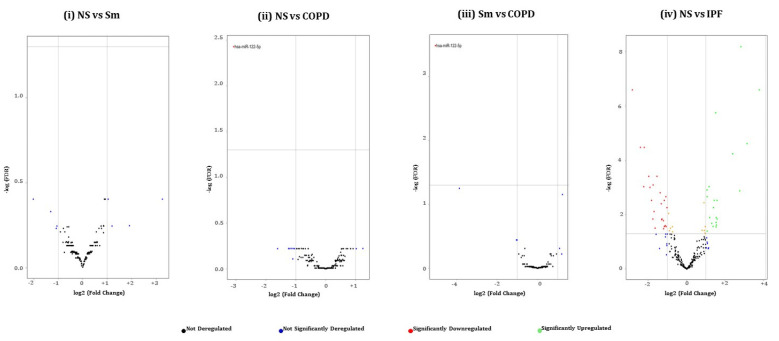
Volcano plots showing number and distribution of miRNAs from lung-tissue-derived exosomes. Volcano plots showing the relation between –log(FDR) (y-axis) vs. log2 (fold change) (x-axis) in the differentially expressed miRNAs amongst lung tissue derived exosomes from (**i**) healthy non-smokers (NS) vs. healthy cigarette smokers (Sm), (**ii**) healthy non-smokers (NS) vs. COPD patients (COPD), (**iii**) healthy cigarette smokers (Sm) vs. COPD patients, and (**iv**) healthy non-smokers (NS) and IPF patients (IPF). To avoid the overlap between the miRNA names, an unlabeled graph for this comparison is used. However, a detailed account of each of the differentially expressed miRNAs is provided in Figure 7.

Next, we generated volcano plots showing pairwise comparisons of the differential miRNA expression profiles between various experimental groups in BALF or lung-tissue-derived exosomes (Figure 4 and Figure 5). We plotted the -log_10_ of adjusted p-values on the y-axis and the log_2_ fold change between two experimental groups on the x-axis to generate a volcano plot. Fold changes greater than ±1 on the logarithmic (base2) scale of the derived volcano plots were considered significant. The miRNAs showing no significant fold change were denoted with blue, while significantly up- or downregulated miRNAs were denoted with green- and red-colored dots respectively.

### 2.4. Hierarchical Clustering Identified Differentially Expressed miRNAs in the BALF or Lung-Tissue-Derived Exosomes from Non-Smokers, Smokers, and Patients of COPD and IPF

**Figure 6:**
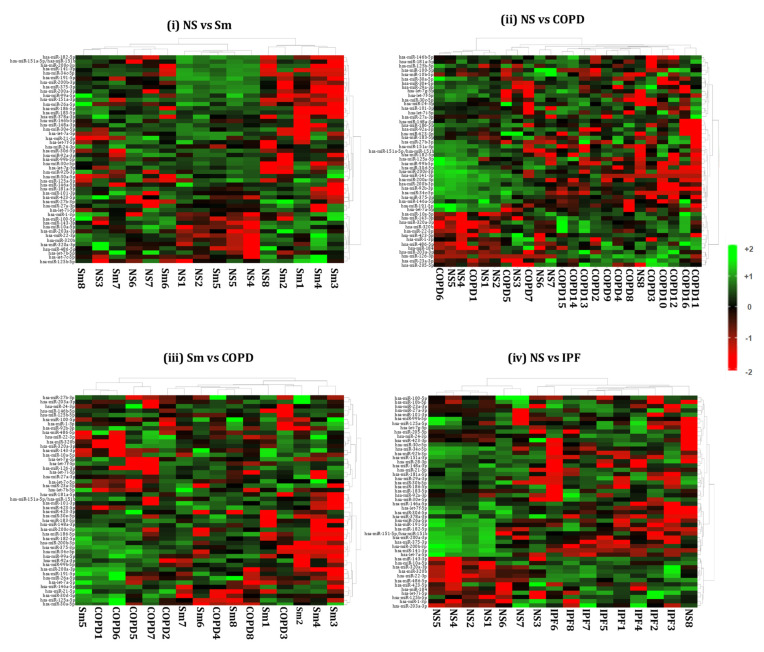
Hierarchical cluster analyses of differentially expressed miRNAs from BALF-derived exosomes. Heat maps showing top 50 variable miRNAs that are differentially expressed in the BALF-derived exosomes from (**i**) healthy non-smokers (NS) vs. healthy smokers (Sm), (**ii**) healthy non-smokers (NS) vs. COPD patients (COPD), (**iii**) healthy smokers (Sm) vs. COPD patients (COPD), and (**iv**) healthy non-smokers (NS) vs. IPF patients (IPF).

**Figure 7:**
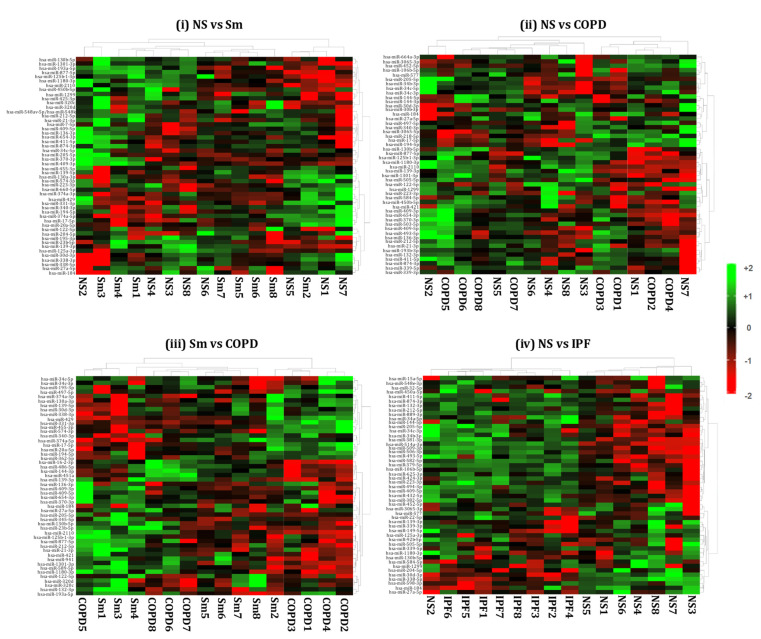
Hierarchical cluster analyses of differentially expressed miRNAs from lung-tissue-derived exosomes. Heat maps showing top 50 variable miRNAs that are differentially expressed in the lung-tissue-derived exosomes from (**i**) healthy non-smokers (NS) vs. healthy smokers (Sm), (**ii**) healthy non-smokers (NS) vs. COPD patients (COPD), (**iii**) healthy smokers (Sm) vs. COPD patients (COPD), and (**iv**) healthy non-smokers (NS) vs. IPF patients (IPF).

We generated heat maps showing the top 50 differentially expressed miRNAs from the BALF- and lung-tissue-derived exosomes from NS vs. Sm, NS vs. COPD, NS vs. IPF, and Sm vs. COPD, as shown in Figure 6 and Figure 7. Each miRNA is depicted in the individual rows of the heat map while the color scale represents the relative expression level as denoted in the scale bar alongside. Detailed information about the significantly altered miRNAs with their respective p-values and biological significance is listed in Supplementary Tables S1 and S2. In brief, the following observations were made when comparing the various experimental pairs: 

**Non-smokers vs. Smokers:** We did not detect any significant differentially expressed miRNAs in the BALF-derived exosomes from smokers and non-smokers. Similarly, no significant variation was observed when comparing the miRNA population from lung-tissue-derived exosomes from smokers and non-smokers.

**Non-smokers vs. COPD:** When comparing the BALF-derived exosomal miRNAs from non-smokers and COPD patients, we found three significant differentially expressed miRNAs. Of these, two (miR-320b and miR-22-3p) were significantly upregulated, while one (miR-423-5p) was significantly downregulated in the BALF-derived exosomes from COPD patients as compared to the non-smoking controls. In contrast, we demonstrated significant downregulation of one (miR-122-5p) exosomal miRNA in the lung tissue of COPD patients as compared to non-smokers.

**Smoker vs. COPD:** We observed significant downregulation of miR-100-5p in the BALF-derived exosomes from COPD patients in comparison to those from healthy smokers. 

Similarly, when comparing the lung-derived exosomes from these two study groups we found a significant downregulation of miR-122-5p in the exosomes derived from the lungs of COPD patients as compared to healthy smokers. Interestingly, the same miRNA was found to be downregulated when comparing the miRNA population from the lung-derived exosomes from COPD patients and non-smokers.

**Non-smokers vs. IPF:** Our results showed a distinct miRNA signature in the BALF- and lung-tissue-derived exosomes from IPF patients as compared to non-smoking controls. Nine differentially expressed miRNAs were identified from the BALF-derived exosomes of IPF patients as compared to healthy non-smoking controls. Of the nine, five (miR-375-3p, miR-200a-3p, miR-200b-3p, miR-141-3p, and miR-423-5p) miRNAs were significantly downregulated, while four (miR-22-3p, miR-320a-3p, miR-320b, and miR-24-3p) were upregulated in the BALF of IPF patients.

Interestingly, we found 55 (26 upregulated and 29 downregulated) differentially expressed miRNAs in the lung-derived exosomes from lungs of IPF patients as compared to non-smoking controls.

**COPD vs. IPF:** We also compared the miRNAs in BALF- and lung-derived exosomes from COPD and IPF patients. We identified one differentially expressed miRNA (miR-375-3p) in BALF-derived exosomes from IPF vs. COPD patients. Likewise, when comparing the differential miRNAs in lung tissue exosomes from COPD and IPF patients, we found 67 significant differentially (31 upregulated; 36 downregulated) expressed miRNAs in COPD patients when compared to IPF disease phenotypes. The detailed plot of these differentially expressed miRNAs and the details of the fold change are available in Supplementary Figures S4 and S5 and Supplementary Table S3.

### 2.5. GO Enrichment and KEGG Analyses of Differentially Expressed miRNAs from BALF- and Lung-Derived Exosomes in COPD and IPF Patients

To understand the potential functions of the differentially expressed miRNAs in COPD and IPF patients, we performed GO enrichment covering three major domains: biological process, cellular compartment, and molecular function. GO term annotation of differentially altered miRNAs in BALF-derived exosomes from COPD patients as compared to healthy non-smokers and smokers resulted in enrichment of terms, including post-translational protein modification, ubiquinone biosynthetic process, cellular component organization, membrane enclosed lumen, clathrin-coated vesicle, mitochondrial matrix, protein binding, protein heterodimerization, and transferase activity. The regulatory pathway annotation via KEGG enrichment analyses showed representation of pathways involved in terpenoid backbone biosynthesis, cAMP signaling, cellular senescence, and chemokine signaling amongst COPD patients. However, none of these pathways was significantly over-represented in our analyses. GO annotation for miRNA populations obtained from IPF patient BALF resulted in enrichment of terms, including lipid transport, mesenchymal cell development, chromatin, mitochondria, lysosome, R-SMAD binding, and ATPase activity. The KEGG analyses for this subject group showed 80% overlap with the pathways enriched amongst COPD patients. Interestingly, however, we found a significant over-representation of pathways regulating glycosaminoglycan biosynthesis (*p* = 0.028) in the BALF-derived exosomes from IPF patients.

GO annotation of differentially regulated miRNAs from lung-derived exosomes was conducted separately. We found enrichment of terms like blood vessel morphogenesis, angiogenesis, transmembrane signaling receptor activity, G-protein-coupled receptor activity, calcium mediated signaling, and calcineurin-NFAT signaling cascade in lung-derived exosomes from COPD patients as compared to healthy individuals (non-smokers and smokers). Contrarily, GO terms, including plasma-membrane-bounded cell projection organization, chemical homeostasis, G-protein-coupled receptor activity, positive regulation of phospholipase C activity, MHC class II protein complex signaling, GTPase activator activity, and positive regulation of non-membrane spanning protein tyrosine kinase activity, were found to be enriched when analyzing differentially expressed miRNAs from lung-derived exosomes in IPF patients. KEGG enrichment analyses showed over-representation of pathways regulating apoptosis, asthma, and the cGMP-PKG signaling pathway, amongst others, in COPD patients. However, none of these regulatory pathways were significantly represented. Contrarily, KEGG enrichment analyses of miRNA profiles from IPF patients identified representation of 40 pathways, of which 12 were significantly represented in the miRNA population from the lung-derived exosomes from IPF patients. 

Table 1, Table 2, Table 3 and Table 4 provide an account of the GO enrichment and KEGG analyses results for our comparisons of various subject groups in this study. Only selected pathways are represented in the Tables.

## 3. Discussion

The role of exosomes in lung diseases has gained increasing attention in recent times due to their role in influencing intercellular communication [10,31]. They are 50–150 nm in diameter, membrane-bound vesicles that contain protein, DNA, mRNAs, microRNAs (miRNA), and small non-coding RNAs to regulate pleiotropic functions. They have been extensively studied with respect to tumor microenvironments and neoplastic cancers and are being studied to be targeted as diagnostic tools or therapy against drug resistance [32,33,34]. However, not much is known about their role in lung pathologies. Recent studies suggest that exosomes mediate cellular crosstalk in lung microenvironments and that cigarette-smoke-induced exosomes promote myofibroblast differentiation in primary lung fibroblasts [35,36]. In addition, activated exosomes (due to cigarette smoke or disease conditions) result in macrophage polarization and matrix destruction in mouse models [37,38]. These studies indicate that exosomes affect cell-to-cell signaling in tobacco-smoke-related disorders.

In this respect, inhalation of toxic agents from tobacco smoke might result in irreparable airway injury, leading to various lung diseases like COPD and IPF. While the etiology/cause of each of these diseases might be due to environmental factors, the disease pathologies are distinct [39]. Therefore, we were interested in understanding if the exosomal population and the exosome-derived miRNA signatures from the BALF and lung tissues of non-smokers, smokers, COPD patients, and IPF patients are unique and can be developed into effective biomarkers for the clinical diagnosis of respective pathologies. Per ISEV guidelines, the isolated exosomes were characterized [30]. Due to the non-availability of a standardized method and variation in the sample quality prior to isolation, we noticed some variations in the purity and quality of the isolated exosomes. However, none of these were below the permissible limits for exosome research. Additionally, further quality checks of the isolated miRNAs were done by Norgen Biotek at the time of sequencing to ascertain successful isolation of good quality miRNAs. We therefore regarded these as exosomes enriched EVs that entail to both the populations derived from BALF and lung tissue. It is also pertinent to mention here that we did not observe any changes in the exosome size or concentration from the BALF or lung tissue of non-smokers, smokers, COPD and IPF patients. No marked difference was found in the RNA or protein concentrations obtained from exosomes isolated from various experimental groups. 

Results from next generation sequencing revealed no significant differentially expressed miRNAs in the BALF- or lung-tissue-derived exosomes from heathy smokers and non-smokers. This suggests that smoking status alone does not affect the exosome-mediated signaling in healthy individuals. However, we found a distinct variation in the miRNA populations from BALF- and lung-tissue-derived exosomes from COPD patients in comparison to healthy non-smokers. We found a three-fold downregulation in the expression of miR-423-5p in the BALF-derived exosomes from COPD patients as compared to healthy non-smoking controls. Of note, miR-423-5p is known to be involved in the regulation of apoptosis and extracellular matrix degradation in human nucleus pulposus cells [40]. Contrary to our findings, Molina-Pinelo et al. (2014) identified increased expression of miR-423-5p in the BALF collected from COPD patients as compared to the control group. However, it is important to mention here that the control group included in this study comprised of a few ex-smokers [41]. Therefore, taken together, it can be concluded that miR-423-5p is crucial in COPD and must be studied further to understand its potential role in the pathophysiology of COPD. 

Further, we observed a two-fold increase in the expression of miR-320b and miR-22-3p in the BALF-derived exosomes from COPD patients as compared to the non-smoking controls. Previous study by our group identified upregulation of both miR-320b and miR-22-3p in the peripheral blood-derived exosomes of COPD patients, thus indicating the significant role of these miRNAs in regulating the disease phenotype. miR-320b is the negative regulator of the mitochondrial mediator, TP53-regulated inhibitor of apoptosis (TRIAP1) and has been previously shown to be upregulated in the peripheral blood mononuclear cells (PBMCs) from COPD patients [42,43]. Similarly, miR-22-3p is reported to inhibit HDAC4 to promote Th17-mediated emphysema in cigarette-smoke (4 month)-exposed C57Bl/6 mice lungs [44]. Serum levels of miR-22-3p are increased amongst COPD patients based on their history of smoking, thus revealing the crucial nature of this miRNA in the progression of the disease [45]. 

When comparing the miRNA expression of lung-tissue-derived exosomes from COPD patients and non-smokers, we observed a three-fold downregulation of miR-122-5p in the lungs of COPD patients as compared to healthy non-smoking controls. Importantly, we further observed a five-fold decrease in the expression of miR-122-5p when comparing the miRNA population from lung-derived exosomes from COPD patients versus healthy smokers. Our results are in accordance with previous literature [46,47,48]. For instance, Zhu et al. (2020) demonstrated the downregulation of miR-122-5p in the sputum and plasma of COPD patients and proved that it functions as a negative regulator of IL-17A production [46]. It is pertinent to mention here that though we did not find any commonly altered miRNAs in the exosomes from the BALF or lung tissue of COPD patients, we found links that associate miRNA-mediated modulation of IL17-signaling amongst the diseased individual. The role of IL-17 in the disease pathology of COPD is rapidly emerging and is known to play an important role in the regulation of chronic inflammation and emphysema/COPD [49,50]. Hence, our findings identify the upstream regulators of this pathway that could possibly alter the IL-17-mediated inflammation in patients with advancing disease.

Next, we identified significant downregulation of miR-100-5p in the BALF-derived exosomes from COPD patients as compared to healthy smokers. Functionally, miR-100 is linked to the regulation of epithelial–mesenchymal transition (EMT), apoptosis, and inflammation [51,52]. Furthermore, Akbas and colleagues have demonstrated downregulation of miR-100-5p in the serum of COPD patients when compared to healthy smokers, which is in accordance with our study results [53]. 

The differentially expressed miRNA population from BALF- and lung-tissue-derived exosomes in COPD and IPF was very distinct in our study. We found five significantly downregulated miRNAs (miR-200a-3p, miR-200b-3p, miR-141-3p, miR-375-3p, and miR-423-3p) and four significantly upregulated miRNAs (miR-320a-3p, miR-320b, miR-22-3p and miR-24-3p) in the BALF-derived exosomes from IPF patients. Of these, miR-423-3p and miR-320b were found to be significantly dysregulated amongst COPD patients as well. Of note, existing reports suggest a role of miR-200 in the pathogenesis of IPF [54,55]. It has been shown that miR-200 promotes TGF-β1-induced EMT in normal cells and its downregulation results in a fibrogenic phenotype in IPF [54]. To our knowledge, there is no existing literature associating miR-141-3p, miR-22-3p, and miR-24-3p with IPF. Thus, we, for the first time, identify the association of these miRNAs with the disease pathogenesis in IPF.

We found 55 differentially expressed miRNAs in the lung-derived exosomes from IPF patients when compared to non-smokers. Of these, many, including miR-514-3p, miR-122-5p, miR-10b-5p, miR-139b-3p, miR-582-5p, miR-889-3p, miR-1-3p, miR-148a-3p, and miR-151b, have never been reported with IPF. Our study, for the first time, reports the correlation of the dysregulated expression of these miRNAs in the lung-derived exosomes from IPF patients. Of note, we observed a three-fold increase in the expression of miR-506-3p in the lung-derived exosomes from IPF patients as compared to the healthy non-smoking controls. Previous work by Zhu et al. (2019) reported that miR-506-3p targets the p65 subunit of NF-κB to induce apoptosis and inflammation in an experimental mice model for IPF. This study concluded that miR-506-3p is a regulator of lung fibrosis [56]. Our results provide clinical evidence suggesting a crucial role of this miRNA in the pathophysiology of IPF in humans. Similarly, accumulating evidence supports the role of miR-21-5p in the disease progression of IPF [57,58,59]. Further, the expression of miR-21-5p is controlled by the levels of TGF-β family proteins and SMADs, both of which are key regulators in the etiology of fibrosis [60]. All these significant differentially expressed miRNAs require validation by qPCR in a larger cohort of the patient population. Since comparing COPD and IPF groups was not the focus of this study, we do not discuss the differentially expressed miRNA populations from both these groups here. Nevertheless, the comparisons for the two disease groups were performed and the heat maps and the fold changes observed for the differentially expressed miRNAs are available in the Supplementary Materials (Supplementary Table S3). Further phenotyping of COPD and IPF will provide information on key miRNAs that are affected in larger cohorts in future studies.

Despite its novelty and relevance to translational implications, our study had some limitations. First, the sample size for each of the study groups was quite small (*n* = 8–16). Second, due to non-availability of age- and gender-matched individuals in our cohorts, we were unable to normalize for the gender and age-specific bias in our results. Age could be a major confounding factor in such a work as the miRNA profiles alter based on aging [61,62,63]. The etiology of both COPD and IPF is affected by age, to which some of the alterations could be attributed to increased cellular senescence and accelerated aging in the diseased individuals. Future work with age- and gender-matched subjects might be able to shed light on this possibility. Third, there was non-availability of non-smokers/never-smokers and limited information regarding the spirometry, pack years, and smoking history of all the subjects included in this study, which may have affected the final interpretation of our findings. 

Overall, this is a first study that compares the BALF- and lung-tissue-derived exosomal miRNAs from IPF and COPD patients with healthy subjects to suggest the unique miRNA signatures that could serve as potential biomarkers to identify the disease progression of these pulmonary conditions and could also be developed as therapeutic targets. Future studies will be designed to validate the findings from this work in larger cohorts and to understand the role of exosomal miRNAs in affecting the disease development, progression, and severity of chronic lung diseases.

## 4. Materials and Methods

### 4.1. Ethics/Approval

The human patients and the patients’ data included in the study were procured from several agencies (described below), as human subject recruitment was not directly involved with this work. The procurement of human lung tissue and BALF samples as de-identified samples was approved by the Materials Transfer Agreement (Institutional Review Board, IRB) with exemption on October 5, 2021 via our RSRB office Study ID: STUDY00006571 is not research involving human subjects as defined by DHHS and FDA regulations, along with laboratory protocols by the Institutional Biosafety Committee (IBC) at the University of Rochester Medical Center, Rochester, NY. The project codes and dates of approval were as follows: project code, DRAI1 001; protocol, 004; date of approval and IRB/IBC approvals, 11 February 2017 and 29 September 2017.

All the procedures/ protocols were carried out per the guidelines and regulations specified by the University of Rochester, Rochester, NY. Other approvals include: (a) IRB study number 20080326 at the University of Miami and (b) registered clinical trial (NCT04016181) ethically approved by the University of Edinburgh (07/S1102/20) and NHS Lothian 2007/R/RES/02 by 14 June 2007. Additional samples were obtained from baseline measurements of Feasibility of Retinoids for the Treatment of Emphysema (FORTE) trial participants, as described previously [64,65].

### 4.2. Study Population and Sample Collection

We employed bronchoalveolar lavage fluid and lung tissue collected from healthy (Non-smokers and Smokers) and diseased (COPD and IPF) human subjects as samples for this study from seven independent cohorts (Table 5). A total of 40 BALF samples and 32 lung tissue samples were chosen for this study from multiple sources. The majority of the BALF samples used in this study were procured from a commercial provider—BioIVT (Westbury, NY, USA). The rest of the BALF samples were provided by our collaborators—Dr. Michael Campos from the Division of Pulmonary, Allergy, Critical Care at the University of Miami, Dr. Haseeb Siddiqi from the Department of Cell Biology at SUNY Downstate Health Sciences University, and Dr. Nikhil Hirani from the Center of Inflammation research at Edinburgh University, UK. The samples procured from our collaborators were validated for their disease categories based on their spirometry and clinical status.

Likewise, the lung samples were procured from three sources: (a) a commercially available resource for procurement of human tissue and organs— the National Disease Research Interchange (NDRI), (b) the NHLBI-funded bio-specimen repository— the Lung Tissue Research Consortium (LTRC), and (c) the Department of Medicine and Pathology at the University of Helsinki Hospital, Finland, as reported previously [66,67]. 

All the subjects included in the study were above 21 years of age. Care was taken to include equal numbers of males and females in each subject group. A detailed characteristic of the BALF and lung tissue samples used for this study is provided in Table 5.

### 4.3. BALF Exosome Isolation

We employed a commercially available Plasma/Serum Exosome Purification and RNA Isolation Midi Kit from Norgen Biotek (Cat# 58500; Norgen Bioteck Corporation, Thorold, ON, Canada) to isolate exosomes from human BALF samples. BALF exosomes were isolated as per the manufacturer’s protocol. In brief, a 1 mL BALF sample was mixed with nuclease-free water, ExoC buffer and Slurry E and incubated for 5 min at room temperature. Next, the solution was centrifuged at 2000 rpm for 5 min at room temperature and the supernatant was discarded. The slurry pellet was then resuspended in ExoR buffer and incubated for 10 min at room temperature. Thereafter, the suspension was centrifuged at 8000 rpm for 2 min at room temperature and transferred to a Mini Filter Spin column to elute the exosomal fraction. The eluted exosomes were then stored at −80 °C until further use.

### 4.4. Lung Tissue Exosome Isolation

The tissue exosomes were isolated using the protocol described by Dooner et al. (2018) [68] with some modifications. In brief, 30–40 mg of lung tissue was chopped and lysed using 1× Liberase solution containing 0.01% DNase. The tube containing tissue lysate was left on an orbital shaker at 37 °C for 1 h to allow complete digestion of lung tissue. After 1 h of incubation, the tissue lysate was collected. The eluate was then centrifuged at 300 g for 10 min at 4 °C to remove cell debris. Next, the supernatant was transferred to a fresh tube and centrifuged at 2000× *g* for 10 min at 4 °C. Again, the supernatant was transferred to a fresh tube and centrifuged at 10,000× *g* for 30 min at 4 °C to remove larger vesicles. Afterward, the supernatant was transferred to ultracentrifuge tubes and the exosomes were pelleted at 110,000× *g* for 70 min at 4 °C using an Optima Max-XP ultracentrifuge (Beckman Coulter, Brea, CA, USA). At this stage, the supernatant was discarded and the pellet was re-suspended in 1× PBS prior to filtering through a 0.22 µM filter. The filtrate was once again spun at 110,000× *g* for 70 min at 4 °C. Finally, the supernatant was discarded and the pellet was re-suspended in 1mL 1× PBS. This contained freshly isolated tissue exosomes that were stored at −80 °C for future analysis.

### 4.5. Exosome Characterization

We employed a Hitachi 7650 analytical transmission electron microscope to visualize the isolated exosomes and nanoparticle tracking analysis (NanoSight NS300) to analyze particle size and concentration, as described earlier [14,69]. 

We also used immunoblotting to identify exosomal markers from the isolated fraction to characterize the BALF- and lung-tissue-derived exosomes. In brief, 20 µg of exosomal lysate was resolved on a 10% sodium dodecyl sulfate (SDS)-polyacrylamide gel and electroblotted onto nitrocellulose membranes. Membranes were blocked using a 5% blocking buffer for 1 h and thereafter probed overnight with antibodies for exosomal surface markers. The antibodies included Histone 4(Cat# 2592) (Cell Signaling Technologies, Danvers, MA, USA), CD9 (Cat# ab92726), CD63 (Cat# ab134045) (Abcam, Waltham, MA, USA), and CD81 (Cat# EXOAB-CD81A-1) (SBI Biosciences, Palo Alto, CA, USA). In the following days, the blots were washed and probed with appropriate secondary antibodies. Chemiluminescence was detected using the Bio-Rad ChemiDoc MP Imaging System using the SuperSignal West Femto Maximum Sensitivity Substrate (Cat# 34096, Thermo Scientific, Waltham, MA, USA).

### 4.6. Exosomal RNA Extraction

Total RNA from BALF exosomes was isolated using an Exosomal RNA Isolation Kit (Cat# 58000, Norgen Bioteck Corporation, Thorold, ON, Canada) as per the manufacturer’s protocol. The detailed procedure has been published earlier by us [13].

Alternately, we used an miRNeasy Mini Kit (Cat# 217004, Qiagen, Germantown, MD, USA) to isolate RNA from lung exosomes as per the manufacturer’s protocol. Briefly, 700 µL of QIAzol lysis buffer was mixed with 250 µL of exosomal fraction and the mix was homogenized using a QIAshredder. The homogenate was then mixed with 140 µL of chloroform to allow phase separation and the aqueous phase was transferred to a fresh tube. Afterward, the RNA was precipitated using 100% ethanol and washed using RWT and RPE buffers provided with the kit. Finally, the RNA was eluted using RNase-free water and stored at −80 °C until further use. The RNA quality and quantity were checked using an Agilent 2100 Bioanalyzer.

### 4.7. Library Preparation

The isolated RNA samples were shipped to Norgen Biotek in Canada for library preparation, sequencing, and data analyses. The library preparation was performed using the standard library preparation workflow of Norgen Biotek, including 3′ and 5′ adapter ligation, followed by reverse transcription, indexing PCR, and size selection using 6% Novex TBE Gel. In brief, a Norgen Biotek Small RNA Library Prep Kit (Cat# 63600, Norgen Bioteck Corporation, Thorold, ON, Canada) was employed for library preparation making sure to use the same lot between each batch of samples. 

Samples were quantified using both PicoGreen and an Agilent 2100 Bioanalyzer. Six µL of high-quality total RNA was mixed with 3′ adaptor and T4 RNA ligase 2 to set up a reaction for 3′ adaptor ligation per the manufacturer’s protocol. This was followed by the removal of excess 3′ adaptor and then 10–12 µL of final eluate was mixed with 5′adaptor to set up a reaction for 5′ adaptor ligation. Next, the reaction for cDNA synthesis was set using the obtained ligated product as input, per the manufacturer’s directions, and incubated at 50 °C for 1 h in a thermocycler. This was followed by PCR amplification and indexing, as advised, and cleanup of final indexed PCR product using an NGS Reaction Cleanup Kit. After cleaning, the samples were run on 6% Novex TBE Gel for 50 min at 140 V. The adaptor dimer not containing any library was excised, and the sample was eluted from the gel and checked for quality as per the manual’s instructions. At this stage, the library quality check was performed to estimate library size and concentration using the bioanalyzer. Samples were then pooled in equimolar ratios and further size-selected using a 3% agarose gel cassette on the Pippin prep (Part # SAG-CDP3010). The pool was quantified by the bioanalyzer before starting the next-generation sequencing (NGS) run.

### 4.8. Next-Generation Sequencing and Data Analysis

We employed a NextSeq 500/550 High Output Kit v2 for 75 cycles (Cat# 20024906, Illumina, San Diego, CA, USA) to perform NGS on our pooled library. Per the manufacturer’s directions, the pooled library was denatured and diluted to the required concentration of 20 pM for optimal cluster generation. The library was then applied onto the suitable flow cell and sequenced using the Illumina NextSeq 500 sequencing platform.

The raw sequence reads were analyzed by the team of bioinformaticians at Norgen Biotek using their advanced analysis pipeline for the processing of raw counts and alignment to the endogenous genome and annotated transcriptome.

### 4.9. Gene Ontology and KEGG Analyses

The gene ontology or GO enrichment analysis [70] was performed through the examination of significant GO terms associated with the differentially expressed miRNAs for each comparison group. The analysis was performed by iteratively testing the enrichment of each annotated GO term correlated with the set of pre-selected differentially expressed genes (in our case, miRNAs) in a linear fashion. Individual enriched annotated GO terms were analyzed using a Fisher’s exact test for both upregulated and downregulated genes in which GO terms with an FDR adjusted p-value threshold of 0.05 were reported as significantly relevant. The FDR is the false discovery rate generated using the Benjamini–Hochberg method, which adjusts the p-value based on the FDR. The analysis was performed separately on all three GO domains, i.e., biological process, molecular function, and cellular component. 

The KEGG enrichment analysis [71] was also performed to identify the differentially expressed genes within an associated pathway for various biological processes. The analysis was performed by testing the enrichment of each biological pathway with the associated gene (or miRNA) found within the set of pre-selected differentially expressed genes. Individually enriched pathways were then contrasted and compared between the two test groups using a Fisher’s exact test for both upregulated and downregulated genes within the pre-selected set of differentially expressed genes. Biological pathways with an adjusted *p*-value below 0.05 were reported.

### 4.10. Statistical Analysis

The miRNA data from various batches were normalized using the trimmed mean of M-values (TMM) normalization method [72]. The TMM normalized read counts were used for differential expression analysis. The principal component analysis (PCA) was plotted using the ggfortify function in the R software (version: 3.5.1) to produce a sample clustering plot based on miRNAs with the highest variation across all samples. The coefficient of variation (%CV) was calculated based on the log_2_ of TMM normalized data and then the 50 miRNAs with the highest %CV were selected and used to generate the PCA plot. The highest two components of variation were plotted on the x-axis (the first principal component, PCA1) and the y-axis (the second principal component, PCA2). Confidence ellipses and average center points were calculated and added for each sample group to further organize the biological groupings.

The EdgeR statistical software package was used for DE analysis, as described previously [73,74]. The Benjamini–Hochberg procedure [75] was then used for adjusting the false discovery rate. This allowed us to identify the significant DE when comparing two groups. The DE was considered significant if a log fold change of ≥1 or ≤−1 at *p*- value and FDR of ≤0.05 was reported for the miRNA target. We used the ggplot2 function in the R software (version: 3.5.1) to plot volcano plots for illustrating a large number of miRNAs and displaying the particular miRNA targets with statistically significant differential expression. 

Heat maps were generated using the ComplexHeatmap function in the R software (version: 3.5.1). The coefficient of variation (%CV) was calculated based on log_2_ of TMM normalized data and then the 50 miRNAs with the highest %CV were selected and used to generate the heat map. 

A Kruskal–Wallis test was used to calculate significance for sample distribution.

## Figures and Tables

**Table 1:** GO enrichment analysis of differentially expressed miRNAs in BALF-derived exosomes.

ID	Term	Ontology	*n*	*p*-Value *
**NS vs. COPD**				
GO:0016043	Cellular component organization	BP	17	0.183535762
GO:0071840	Cellular component organization or biogenesis	BP	17	0.183535762
GO:0061024	Membrane organization	BP	3	0.149797571
GO:0051259	Protein complex oligomerization	BP	3	0.149797571
GO:0006720	Isoprenoid metabolic process	BP	2	0.101214575
GO:0051186	Co-factor metabolic process	BP	2	0.101214575
GO:0051188	Co-factor biosynthetic process	BP	2	0.101214575
GO:0051262	Protein tetramerization	BP	2	0.101214575
GO:0008299	Isoprenoid biosynthetic process	BP	2	0.101214575
GO:0006732	Co-enzyme metabolic process	BP	1	0.051282051
GO:0009108	Co-enzyme biosynthetic process	BP	1	0.051282051
GO:0006733	Oxidoreduction coenzyme metabolic process	BP	1	0.051282051
GO:0043687	Post-translational protein modification	BP	1	0.051282051
GO:1901661	Quinone metabolic process	BP	1	0.051282051
GO:0042181	Ketone biosynthetic process	BP	1	0.051282051
GO:0051290	Protein heterotetramerization	BP	1	0.051282051
GO:0051291	Protein heterooligomerization	BP	1	0.051282051
GO:0006743	Ubiquinone metabolic process	BP	1	0.051282051
GO:0006744	Ubiquinone biosynthetic process	BP	1	0.051282051
GO:1901663	Quinone biosynthetic process	BP	1	0.051282051
GO:0031974	Membrane-enclosed lumen	CC	11	0.007422402
GO:0070013	Intracellular organelle lumen	CC	11	0.007422402
GO:0030659	Cytoplasmic vesicle membrane	CC	5	0.24291498
GO:0044431	Golgi apparatus part	CC	5	0.24291498
GO:0044429	Mitochondrial part	CC	3	0.149797571
GO:1902494	Catalytic complex	CC	3	0.149797571
GO:1990234	Transferase complex	CC	2	0.101214575
GO:0005788	Endoplasmic reticulum lumen	CC	2	0.101214575
GO:0030133	Transport vesicle	CC	2	0.101214575
GO:0005802	Trans-Golgi network	CC	1	0.051282051
GO:0030135	Coated vesicle	CC	1	0.051282051
GO:0030136	Clathrin-coated vesicle	CC	1	0.051282051
GO:0030662	Coated vesicle membrane	CC	1	0.051282051
GO:0030665	Clathrin-coated vesicle membrane	CC	1	0.051282051
GO:0005759	Mitochondrial matrix	CC	1	0.051282051
GO:0016765	Transferase activity, transferring alkyl, or aryl (other than methyl) groups	MF	2	0.101214575
GO:0046982	Protein heterodimerization	MF	1	0.051282051
GO:0000010	Trans-hexaprenyltranstransferase activity	MF	1	0.051282051
GO:0050347	Trans-octaprenyltranstransferase activity	MF	1	0.051282051
**Sm vs. COPD**				
GO:0007623	Circadian rhythm	BP	4	0.114285714
GO:0048511	Rhythmic process	BP	4	0.114285714
GO:1901566	Organonitrogen compound biosynthetic process	BP	4	0.114285714
GO:1901135	Carbohydrate derivative metabolic process	BP	3	0.085714286
GO:1901137	Carbohydrate derivative biosynthetic process	BP	2	0.057142857
GO:0006022	Aminoglycan metabolic process	BP	1	0.028571429
GO:0006023	Aminoglycan biosynthetic process	BP	1	0.028571429
GO:0006024	Glycosaminoglycan biosynthetic process	BP	1	0.028571429
GO:0005794	Glycosaminoglycan metabolic process	BP	1	0.028571429
GO:0030203	Bounding membrane of organelle	CC	8	0.228571429
GO:0001904	Organelle sub-compartment	CC	6	0.171428571
GO:0044431	Golgi apparatus	CC	6	0.171428571
GO:0098588	Golgi sub-compartment	CC	5	0.142857143
GO:0098791	Golgi membrane	CC	4	0.114285714
GO:0016740	Transferase activity	MF	5	0.142857143
GO:0016782	Transferase activity, transferring sulfur-containing groups	MF	1	0.028571429
GO:0008146	Sulfotransferase activity	MF	1	0.028571429
GO:0034483	Heparan sulfate sulfotransferase activity	MF	1	0.028571429
GO:0033871	(Heparan sulfate)-glucosamine-3-sulfotransferase-2-activity	MF	1	0.028571429
**NS vs. IPF**				
GO:0009636	Response to toxic substance	BP	2	0.142682927
GO:0097324	Melanocyte migration	BP	1	0.073170732
GO:0097324	Melanosome organization	BP	1	0.073170732
GO:0014031	Mesenchymal cell development	BP	1	0.073170732
GO:0034204	Lipid transport	BP	1	0.073170732
GO:0044429	Mitochondrial part	CC	3	0.010787992
GO:0005739	Mitochondrion	CC	5	0.034709193
GO:0030136	Clathrin-coated vesicle	CC	1	0.073170732
GO:0000785	Chromatin	CC	1	0.073170732
GO:0005766	Primary lysosome	CC	1	0.073170732
GO:0000010	Trans-hexaprenyltranstransferase activity	MF	1	0.073170732
GO:0050347	Trans-octaprenyltranstransferase activity	MF	1	0.073170732
GO:0016887	ATPase activity	MF	1	0.073170732
GO:0070412	R-SMAD binding	MF	1	0.073170732

* *p*-value for genes that were significantly up- or downregulated.

**Table 2:** GO enrichment analysis of differentially expressed miRNAs in lung-tissue-derived exosomes.

ID	Term	Ontology	*n*	*p*-Value *
**NS vs. COPD**				
GO:0048514	Blood vessel morphogenesis	BP	12	0.068181818
GO:0050808	Synapse organization	BP	11	0.0625
GO:0051962	Positive regulation of nervous system development	BP	11	0.0625
GO:0044089	Positive regulation of cellular component biogenesis	BP	10	0.056818182
GO:0044430	Cytoskeletal part	CC	9	0.051136364
GO:0001525	Angiogenesis	BP	8	0.045454545
GO:0050803	Regulation of synapse structure or activity	BP	8	0.045454545
GO:0050807	Regulation of synapse organization	BP	8	0.045454545
GO:0015630	Microtubule cytoskeleton	CC	8	0.045454545
GO:0038023	Signaling receptor activity	MF	8	0.045454545
GO:0060089	Molecular transducer activity	MF	8	0.045454545
GO:0004888	Transmembrane signaling receptor activity	MF	7	0.039772727
GO:0004930	G-protein-coupled receptor activity	MF	7	0.039772727
GO:0019932	Second-messenger-mediated signaling	BP	6	0.034090909
GO:0007416	Synapse assembly	BP	6	0.034090909
GO:0045765	Regulation of angiogenesis	BP	5	0.028409091
GO:1901342	Regulation of vasculature development	BP	5	0.028409091
GO:0051963	Regulation of synapse assembly	BP	5	0.028409091
GO:0051965	Positive regulation of synapse assembly	BP	4	0.022727273
GO:0019722	Calcium-mediated signaling	BP	4	0.022727273
GO:0005815	Microtubule organizing center	CC	4	0.022727273
GO:0005813	Centrosome	CC	4	0.022727273
GO:0016525	Negative regulation of angiogenesis	BP	3	0.017045455
GO:1901343	Negative regulation of vasculature development	BP	3	0.017045455
GO:2000181	Negative regulation of blood vessel morphogenesis	BP	3	0.017045455
GO:0033173	Calcineurin-NFAT signaling cascade	BP	2	0.011363636
GO:0048016	Inositol-phosphate-mediated signaling	BP	2	0.011363636
GO:0097720	Calcineurin-mediated signaling	BP	2	0.011363636
**Sm vs. COPD**				
GO:0048514	Blood vessel morphogenesis	BP	11	0.071428571
GO:0044087	Regulation of cellular component biogenesis	BP	11	0.071428571
GO:0044089	Positive regulation of cellular component biogenesis	BP	10	0.064935065
GO:0051962	Positive regulation of nervous system development	BP	10	0.064935065
GO:0001525	Angiogenesis	BP	8	0.051948052
GO:0050803	Regulation of synapse structure or activity	BP	8	0.051948052
GO:0050807	Regulation of synapse organization	BP	8	0.051948052
GO:0044430	Cytoskeletal part	CC	7	0.045454545
GO:0038023	Signaling receptor activity	MF	7	0.045454545
GO:0060089	Molecular transducer activity	MF	7	0.045454545
GO:0019932	Second-messenger-mediated signaling	BP	6	0.038961039
GO:0007416	Synapse assembly	BP	6	0.038961039
GO:0015630	Microtubule cytoskeleton	CC	6	0.038961039
GO:0004888	Transmembrane signaling receptor activity	MF	6	0.038961039
GO:0045765	Regulation of angiogenesis	BP	5	0.032467532
GO:1901342	Regulation of vasculature development	BP	5	0.032467532
GO:0051963	Regulation of synapse assembly	BP	5	0.032467532
GO:0051965	Positive regulation of synapse assembly	BP	4	0.025974026
GO:0019722	Calcium-mediated signaling	BP	4	0.025974026
GO:0016525	Negative regulation of angiogenesis	BP	3	0.019480519
GO:1901343	Negative regulation of vasculature development	BP	3	0.019480519
GO:2000181	Negative regulation of blood vessel morphogenesis	BP	3	0.019480519
GO:0005815	Microtubule organizing center	CC	3	0.019480519
GO:0005813	Centrosome	CC	3	0.019480519
GO:0004930	G-protein-coupled receptor activity	MF	3	0.019480519
GO:0033173	Calcineurin-NFAT signaling cascade	BP	2	0.012987013
GO:0048016	Inositol-phosphate-mediated signaling	BP	2	0.012987013
GO:0097720	Calcineurin-mediated signaling	BP	2	0.012987013
**NS vs. IPF**				
GO:0065008	Regulation of biological quality	BP	44	0.0327192
GO:0007399	Nervous system development	BP	36	0.006858696
GO:0048878	Chemical homeostasis	BP	18	0.003415
GO:0030030	Cell projection organization	BP	17	0.012599483
GO:0120036	Plasma-membrane-bounded cell projection organization	BP	17	0.01259943
GO:0044459	Plasma membrane region	CC	10	0.028105097
GO:0007416	Synapse assembly	BP	7	0.006212841
GO:0030424	Axon	CC	7	0.0488352
GO:0150034	Distal axon	CC	6	0.0305685
GO:0031349	Positive regulation of defense response	BP	5	0.016747
GO:0044306	Neuron projection terminus	CC	4	0.007341699
GO:0008092	Cytoskeletal protein binding	MF	4	0.0073417
GO:0004930	G-protein-coupled receptor activity	MF	4	0.083811139
GO:0051965	Positive regulation of synapse assembly	BP	4	0.007341699
GO:0010863	Positive regulation of phospholipase C activity	BP	2	0.01653348
GO:0043235	Receptor complex	CC	2	0.01653348
GO:0023026	MHC class II protein complex binding	MF	2	0.0165335
GO:0005096	GTPase activator activity	MF	2	0.0165335
GO:1903997	Positive regulation of non-membrane spanning protein tyrosine kinase activity	BP	2	0.01653348

* *p*-value for genes that were significantly up- or down-regulated. BP = biological process; CC = cellular component, and MF = molecular function.

**Table 3:** KEGG analyses of differentially expressed miRNAs in BALF-derived exosomes from COPD and IPF patients.

KEGG Pathway	Selected Pathway
**COPD** ^ƚ^	
path:hsa00900	Terpenoid backbone biosynthesis
path:hsa04920	Adipocytokine signaling pathway
path:hsa00520	Amino sugar and nucleotide sugar metabolism
path:hsa04152	AMPK signaling pathway
path:hsa04371	Apelin signaling pathway
path:hsa04140	Autophagy-animal
path:hsa04136	Autophagy-other
path:hsa01040	Biosynthesis of unsaturated fatty acids
path:hsa04024	cAMP signaling pathway
path:hsa04218	Cellular senescence
path:hsa04062	Chemokine signaling pathway
path:hsa00534 *	Glycosaminoglycan biosynthesis
**IPF** ^#^	
path:hsa00900	Terpenoid backbone biosynthesis
path:hsa04920	Adipocytokine signaling pathway
path:hsa00520	Amino sugar and nucleotide sugar metabolism
path:hsa04371	Apelin signaling pathway
path:hsa04140	Autophagy-animal
path:hsa04136	Autophagy-other
path:hsa01040	Biosynthesis of unsaturated fatty acids
path:hsa04218	Cellular senescence
path:hsa04062	Chemokine signaling pathway
path: hsa05206	microRNAs in cancer

* Significantly enriched pathway; ^ƚ^ COPD patients vs. healthy controls (non-smokers and smokers); ^#^ IPF patients vs. healthy non-smokers.

**Table 4:** KEGG analyses of differentially expressed miRNAs in lung-tissue-derived exosomes from COPD and IPF patients.

KEGG Pathway	Selected Pathway
**COPD** ^ƚ^	
path:hsa04520	Adherens junction
path:hsa04920	Adipocytokine signaling pathway
path:hsa04261	Adrenergic signaling in cardiomyocytes
path:hsa04960	Aldosterone-regulated sodium reabsorption
path:hsa00520	Amino sugar and nucleotide sugar metabolism
path:hsa04215	Apoptosis—multiple species
path:hsa05310	Asthma
path:hsa05100	Bacterial invasion of epithelial cells
path:hsa01040	Biosynthesis of unsaturated fatty acids
path:hsa04260	Cardiac muscle contraction
path:hsa04022	cGMP-PKG signaling pathway
**IPF** ^#^	
path:hsa04972 *	Pancreatic secretion
path:hsa04970 *	Salivary secretion
path:hsa04911 *	Insulin secretion
path:hsa05416 *	Viral myocarditis
path:hsa05310	Asthma
path:hsa01040	Biosynthesis of unsaturated fatty acids
path:hsa04022	cGMP-PKG signaling pathway
path:hsa04014 *	Ras signaling pathway
path:hsa04727 *	GABAergic synapse
path:hsa05033 *	Nicotine addiction
path:hsa04722 *	Neurotrophin signaling pathway
path:hsa04010 *	MAPK signaling pathway
path:hsa04151	PI3K-Akt signaling pathway

* Significantly enriched pathway; ^ƚ^ COPD patients vs. healthy controls (non-smokers and smokers); ^#^ IPF patients vs. healthy non-smokers.

**Table 5:** Clinical characteristics of study subjects.

Characteristics	Non-Smokers	Smokers	COPD/Emphysema	IPF	*p*-Value *
***BALF***					
N	8	8	16	8	
Age (years), mean (SD)	49.6 (17.3)	57.4 (8.9)	65.9 (13.3)	76.5 (11.4)	0.0029
Gender					0.2952
Male n (%)	3 (37.5)	2 (25)	7 (38.9)	6 (75)	
Not specified	0	0	2	0	
Smoking status					0.9719
Current smoker	0	6	2	0	
Ex-smoker	0	2	6	4	
N/A	0	0	7	0	
***Lung Tissue***					
N	8	8	8	8	
Age (years), mean (SD)	48.3 (16.3)	53.8 (15.4)	59.1 (9.9)	68.9 (9.6)	0.0688
Gender					0.981
Male n (%)	4 (50)	6 (75)	3 (37.5)	5 (62.5)	
N/A	2	1	0	0	
Smoking status					>0.9999
Current smoker	0	7	2	0	
Ex-smoker	3	1	4	6	
N/A	0	0	1	0	

*: Kruskal–Wallis test.

## Data Availability

All the data included in this manuscript is available online and is free to access to all readers. The NGS data and/or analyzed files during the current study are available at Gene Expression Omnibus accession number GSE180651 (https://www.ncbi.nlm.nih.gov/geo/query/acc.cgi?acc = GSE180651). All authors confirm the availability of data and materials is online/free access to readers. The manuscript is available as a pre-print on the Preprint and medRxiv servers.

## Supplementary Materials

Supplementary data is available online at https://www.mdpi.com/article/10.3390/ijms222111830/s1.

## Abbreviations

COPD	Chronic obstructive pulmonary disease
IPF	Idiopathic pulmonary disease
TEM	Transmission electron microscopy
NGS	Next-generation sequencing
miRNA	Micro RNA
BALF	Bronchoalveolar lavage fluid
EMT	Epithelial–mesenchymal transition
ISEV	International Society for Extracellular Vesicles
