# Supplementary material for: Distinct Exosomal miRNA Profiles from BALF and Lung Tissue of COPD and IPF Patients
Source: Int J Mol Sci. 2021 Oct 31;22(21):11830. doi: 10.3390/ijms222111830 (PMC8584050; doi:10.3390/ijms222111830)
Supplement: Supplementary file 1 [file ijms-22-11830-s001.zip › ijms-1374816-supplementary.pdf]

Supplementary Information

Distinct exosomal miRNA profiles from BALF and lung tissue from COPD and IPF patients

Gagandeep Kaur[1], Krishna Prahlad Maremanda[1], Michael Campos[2], Hitendra S Chand[3], Feng Li[4], Nikhil Hirani[4], M.A. Haseeb[5,] Dongmei Li[6], and Irfan Rahman[1*]

Supplementary Tables

Supplementary Table S1: Significant Differentially expressed miRNAs in BALF exosomes.
derived

miRNA	log2foldchange	logCPM	pvalue	FDR	Functional Annotation
**NS vs COPD**					
hsa-miR-423-5p	-3.389204282	14.75498	0.00013369	0.0066	mRNA binding involved in posttranscriptional gene silencing. Role in apoptosis and cell proliferation
hsa-miR-320b	2.638473041	14.15853	0.00098978	0.01815	tumor suppressing role by targeting CDK6
hsa-miR-22-3p	2.508785494	18.34373	0.00023988	0.0066	negative regulator of endothelial cell proliferation and necroptotic process; but positive regulator of inflammatory response
**Sm vs COPD**					
hsa-miR-100-5p	-3.492285687	18.86802	3.29E-05	0.00158	negative regulation of cell differentiation and IL-8 secretion
**NS vs IPF**					
hsa-miR-200a-3p	-4.811568605	13.79069	7.88E-06	0.00014	positive regulator of blood vessel endothelial migration, role in Endothelial-mesenchymal transition
hsa-miR-141-3p	-4.31928888	11.4448	0.00045173	0.00422	positive regulator of cell proliferation and negative regulator of leukocyte adhesion to vascular endothelial cell

hsa-miR-375-3p	-4.255714743	12.47332	1.28E-05	0.00014	RNA polymerase II binding complex; positive regulator of endothelial cell apoptotic process
hsa-miR-423-5p	-3.37545635	14.95874	0.00376795	0.02416	mRNA binding involved in posttranscriptional gene silencing. Role in apoptosis and cell proliferation
hsa-miR-320b	3.186119102	14.24854	3.22E-06	9.03E-05	tumor suppressing role by targeting CDK6
hsa-miR-200b-3p	-2.849476778	14.33963	1.21E-05	0.00014	positive regulator of cell proliferation; negative regulator of gene expression
hsa-miR-22-3p	2.817552446	18.23789	3.10E-06	9.03E-05	negative regulator of endothelial cell proliferation and necroptotic process; but positive regulator of inflammatory response
hsa-miR-320a-3p	1.906370214	14.44337	0.00267282	0.02138	negative regulator of cell proliferation, cell migration and IL-4 biosynthetic process.
hsa-miR-24-3p	1.83111351	12.04935	0.00388364	0.02416	negative regulator of angiogenesis and blood vessel endothelial cell migration

<u>Supplementary Table S2:</u> Significant differentially expressed miRNAs in Lung-derived exosomes.

miRNA	log2foldchange	logCPM	pvalue	FDR	Function
**NS vs COPD**					
hsa-miR-122-5p	-3.081717764	8.0592	1.66E-05	0.00374	cell proliferation, migration, invasion, and EMT
**Sm vs COPD**					

hsa-miR-122-5p	-5.048802634	9.96494	1.64E-06	0.00033	cell proliferation, migration, invasion, and EMT
**NS vs IPF**					
hsa-miR-514a-3p	3.779748499	4.04505	3.15E-09	2.56E-07	Tumor suppressor
hsa-miR-506-3p	3.146428357	5.6144	4.98E-07	2.43E-05	negative regulator of cellular biosynthetic pathwaya and IL8 secretion
hsa-miR-21-5p	2.828829977	15.6156	2.70E-11	6.58E-09	negative modulator of angiogenesis; regulates extracellular matrix degradation
hsa-miR-338-5p	-2.821754174	8.55174	2.59E-09	2.56E-07	negative regulation of IL6 secretion and cell migration
hsa-miR-509-3p	2.767633188	5.4511	8.72E-05	0.00133	negative regulator of cell migration, invasion and proliferation
hsa-miR-223-3p	2.402273957	8.20475	1.88E-06	5.74E-05	negative regulator of GTPase activity and regulated macrophage differentiation
hsa-miR-30d-5p	-2.400812469	16.3117	8.89E-07	3.35E-05	regulates gene expression and EMT
hsa-miR-30d-3p	-2.22958366	5.29936	4.73E-05	0.00094	inhibitor of cell proliferation and invasion
hsa-miR-338-3p	-2.204598086	7.29224	9.62E-07	3.35E-05	negative regulator of cell migration and IL6 production
hsa-miR-30b-5p	-1.967255645	12.7584	1.59E-05	0.00039	positive regulator of TGF-beta signaling pathway, regulates lipid metabolism, association with senescence
hsa-miR-204-5p	-1.904426753	5.64924	5.69E-05	0.00099	negative regulation of IL6, IL1beta production and cell migration, regulates EMT
hsa-miR-122-5p	-1.822688383	8.34723	0.00025	0.00299	cell proliferation, migration, invasion, and EMT
hsa-miR-10b-5p	-1.754493357	13.4712	0.00202	0.01452	positive regulator of cell migration, Th17 regulator
hsa-miR-203a-3p	-1.740223733	10.5438	3.65E-05	0.00081	negative regulator of IL8 secretion, regulates EMT
hsa-miR-139-3p	-1.692253283	4.65751	0.00088	0.0077	Tumor suppressor, EMT

hsa-miR-486-5p	-1.636372182	13.0762	0.00604	0.03137	regulates gene expression and has been shown to be associated with IPF
hsa-miR-582-5p	1.590929655	3.94706	0.00196	0.01452	tumor suppressor
hsa-miR-21-3p	1.582268505	7.57847	0.00027	0.00299	negative modulator of angiogenesis; regulates extracellular matrix degradation
hsa-miR-379-5p	1.553371104	4.88324	0.00385	0.02409	negative regulator of cell proliferation and positive regulator of apoptosis
hsa-miR-132-3p	1.543109071	5.19083	0.00161	0.0128	negative regulator of gene expression, regulates inflammation and cell proliferation
hsa-miR-224-5p	-1.541206326	9.28135	1.55E-05	0.00039	regulates cell proliferation, migration and invasion through PIK3R3/Akt pathway
hsa-miR-144-5p	1.53599799	5.70304	0.00293	0.01932	negative regulator of EMT; positive regulation of mitochondrial organization
hsa-miR-889-3p	1.534772448	4.45827	0.00534	0.02833	regulates cell viability and invasion
hsa-miR-142-5p	1.514303487	8.17025	2.93E-08	1.79E-06	negative regulation of IL1beta, response to TNF
hsa-miR-7-5p	1.436816898	5.84014	0.00027	0.00299	inhibits cell migration and invasion, suppresses tumor metastasis
hsa-miR-1-3p	1.398003997	9.02882	0.00059	0.00551	cell proliferation and invasion
hsa-miR-424-3p	1.377444275	3.95708	0.00457	0.02718	negative regulation of cell proliferation and invasion, negative regulation of fibroblast growth factor receptor signaling
hsa-miR-10a-5p	-1.373153961	15.8906	0.00011	0.00157	cell migration and endothelial growth factor signaling
hsa-miR-125a-5p	-1.318243293	14.1273	0.00041	0.00397	fibroblast transdifferentiation, negative regulation of IL16
hsa-miR-106b-5p	1.308396822	4.51807	0.00325	0.02087	regulates EMT and TGF beta signaling, negative regulator of IL8
hsa-miR-23b-5p	-1.300249004	5.24808	0.00219	0.01524	TGF beta signaling regulation
hsa-miR-628-3p	-1.275911117	6.31838	0.00199	0.01452	regulator of cell proliferator

hsa-miR-181d-5p	-1.204679475	6.05627	0.00246	0.01669	inhibitor of cell proliferation and metastasis
hsa-miR-155-5p	1.199631772	7.4596	0.00163	0.0128	negative regulator of cytokine secretion and regulates epithelial barrier and EMT
hsa-miR-197-3p	-1.197907775	5.53844	0.00663	0.03303	negative regulation of IL-18
hsa-miR-183-5p	1.168387995	11.1446	4.99E-05	0.00094	TGF-beta signaling, positive regulation of phagocytosis
hsa-miR-126-5p	-1.161697617	10.4425	0.00028	0.00299	negative regulator of cell migration
hsa-miR-423-5p	-1.158386165	11.3123	0.00472	0.02741	negative regulator of cell invasion and migration
hsa-miR-589-5p	-1.113954446	5.58091	0.00416	0.0254	regulates EMT
hsa-miR-30a-5p	-1.077861429	14.4068	0.00017	0.00219	regulator of TGF-beta signaling
hsa-miR-144-3p	1.07056553	7.30821	0.00874	0.04023	negative regulator of EMT and positive regulator of mitochondrial organization
hsa-miR-199a-3p|hsa-miR-199b-3p	1.068687434	12.3784	0.00017	0.00219	positive regulator of endothelial cell migration, negative regulation of autophagy
hsa-miR-182-5p	1.0640442	11.2871	7.58E-05	0.00123	positive regulator of cell migration and cytokine secretion
hsa-miR-92a-3p	-1.061586028	11.5755	0.00486	0.02742	regulates TGF-beta signalling
hsa-miR-126-3p	-1.020449014	12.1275	0.00061	0.00551	role in angiogenesis and vascular homeostasis
hsa-miR-340-5p	0.968709958	7.35372	0.00494	0.02742	regulates cell proliferation
hsa-miR-409-3p	0.957771401	6.15189	0.01087	0.04821	promotes tumorigenesis
hsa-miR-30a-3p	-0.932099425	10.06	0.00107	0.00904	negative regulator of EMT
hsa-miR-146b-5p	0.920971946	15.3479	0.00797	0.03815	negative regulator of endothelial activation, EMT and IL17 pathway
hsa-miR-148a-3p	0.903657387	12.9195	0.00036	0.00362	role in tumor suppression
hsa-miR-342-3p	-0.890916332	9.21854	0.00834	0.03915	regulates TGF-beta signaling
hsa-miR-151b	-0.840780828	7.66417	0.0062	0.03153	represses cell migration
hsa-miR-103a-3p	0.80597023	9.14838	0.00769	0.03754	negative regulation of peptidyl-threonine

					phosphorylation, regulates glucose homeostasis
hsa-miR-186-5p	-0.734165882	11.605	0.00512	0.02778	regulates collagen and EMT
hsa-miR-151a-5p|hsa-miR-151b	-0.72411126	12.6144	0.00951	0.04297	regulates cell proliferation, migration and invasion

<u>Supplementary Table S3</u>: Significant differentially expressed miRNAs on comparing the miRNAs derived from BALF or Lung-derived exosomes from IPF vs COPD patients.

miRNAs	log2FoldChange	pvalue
**BALF**		
hsa-miR-375-3p	3.699215206	9.33E-05
**Lung Tissues**		
hsa-miR-10a-5p	1.409527466	1.74E-06
hsa-miR-99b-5p	0.849983751	0.0006781
hsa-miR-125a-5p	1.491008563	7.53E-05
hsa-miR-181a-5p	0.64855263	0.0039477
hsa-miR-21-5p	-2.735601026	1.21E-09
hsa-miR-125b-5p	0.928588863	0.0002024
hsa-miR-23a-3p	0.856645231	0.0034096
hsa-miR-199a-3p|hsa-miR-199b-3p	-0.816065829	0.0042361
hsa-miR-151a-5p|hsa-miR-151b	0.80742856	0.0024248
hsa-miR-92a-3p	1.120016981	0.0023208
hsa-miR-182-5p	-1.034019544	0.0002701
hsa-miR-221-5p	0.751510707	0.0041159
hsa-miR-145-5p	0.792259796	0.0053713
hsa-miR-342-3p	1.28928554	1.01E-05
hsa-miR-103a-3p	-0.886227511	0.0037171
hsa-miR-224-5p	1.033150905	0.0025444
hsa-miR-338-5p	2.10636214	2.35E-06
hsa-miR-338-3p	1.324111402	0.0046041
hsa-miR-429	-1.35855317	0.0056567
hsa-miR-1-3p	-1.341960391	0.000599
hsa-miR-142-5p	-1.323394065	4.86E-08
hsa-miR-484	0.875965255	0.0053921
hsa-miR-151b	0.813176399	0.0020734
hsa-miR-340-5p	-1.386800711	9.40E-06

hsa-miR-223-3p	-3.347367864	1.96E-14
hsa-miR-374a-5p	-1.512435161	7.90E-05
hsa-miR-1271-5p	1.294527177	0.000127
hsa-miR-374a-3p	-1.32118481	0.0002536
hsa-miR-365a-3p|hsa-miR-365b-3p	1.255379081	0.0005726
hsa-miR-181d-5p	1.006691821	0.0032183
hsa-miR-205-5p	-1.744538429	0.0032418
hsa-miR-197-3p	1.883112149	1.55E-08
hsa-miR-21-3p	-1.491535768	0.0055195
hsa-miR-204-5p	1.232430302	0.0020586
hsa-miR-345-5p	-1.251470622	0.0001833
hsa-miR-23b-5p	1.489623445	0.0005267
hsa-miR-107	-0.870195285	0.0056404
hsa-miR-32-5p	-1.49781331	0.0038593
hsa-miR-139-3p	1.549991658	0.0003531
hsa-miR-24-2-5p	-1.705632668	0.0002179
hsa-miR-328-3p	1.448057593	0.0013465
hsa-miR-22-5p	-1.413680584	0.003023
hsa-miR-142-3p	-2.681623009	2.97E-05
hsa-miR-548e-3p	-1.185559978	0.0033579
hsa-miR-424-5p	-2.000054742	0.0038114
hsa-miR-889-3p	-1.595329766	0.0022859
hsa-miR-135b-5p	-1.860676506	0.0033555
hsa-miR-381-3p	-1.47381252	0.0014309
hsa-miR-3613-3p	2.375696693	0.0035104
hsa-miR-223-5p	-3.145005297	0.0001399
hsa-miR-3613-5p	-3.686449059	4.50E-07
hsa-miR-508-3p	-2.637539744	0.0035568
hsa-miR-708-5p	-2.487002959	0.0011469
hsa-miR-3130-3p	-3.482486124	0.0013965
hsa-miR-31-5p	-3.475504137	2.37E-05
hsa-miR-377-3p	-3.248729824	3.06E-06
hsa-miR-514a-3p	-2.611946541	0.0014603
hsa-miR-369-3p	-2.604592827	0.0020431
hsa-miR-4732-3p	4.179938703	0.0038075
hsa-miR-449a	-3.994764171	0.0025207
hsa-miR-4683	4.884780183	1.98E-05
hsa-miR-1291	2.949952436	0.0031089
hsa-miR-7977	4.359794794	1.89E-05
hsa-miR-656-3p	-2.054137869	0.0051107
hsa-miR-4738-3p	4.411823484	0.0001546

hsa-miR-6892-5p	2.760729508	0.0036942
hsa-miR-1277-5p	-5.967010493	0.0010824

Supplementary Figures

Uncut Original Blots

Full unedited gel for Figure 1(iii)

Exosome Markers CD9 and CD81 in BALF exosomes

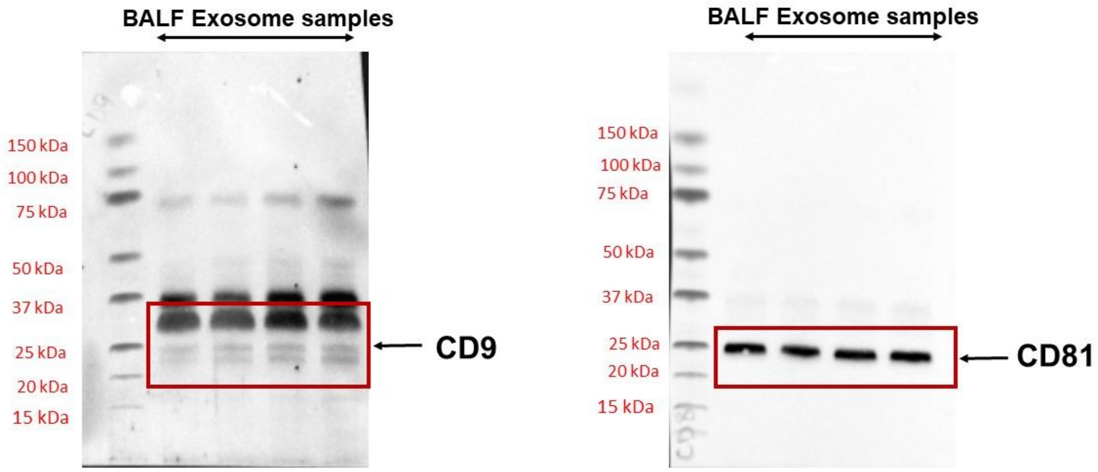

Supplementary Figure S1: Figure showing the original uncut blots for CD9 and CD81from BALF exosome fraction as shown in Figure 1(iii) of the manuscript.

Uncut Original Blots

Full unedited gel for Figure 2(iii)

Exosome Markers CD63 and CD81 in Lung tissue exosomes

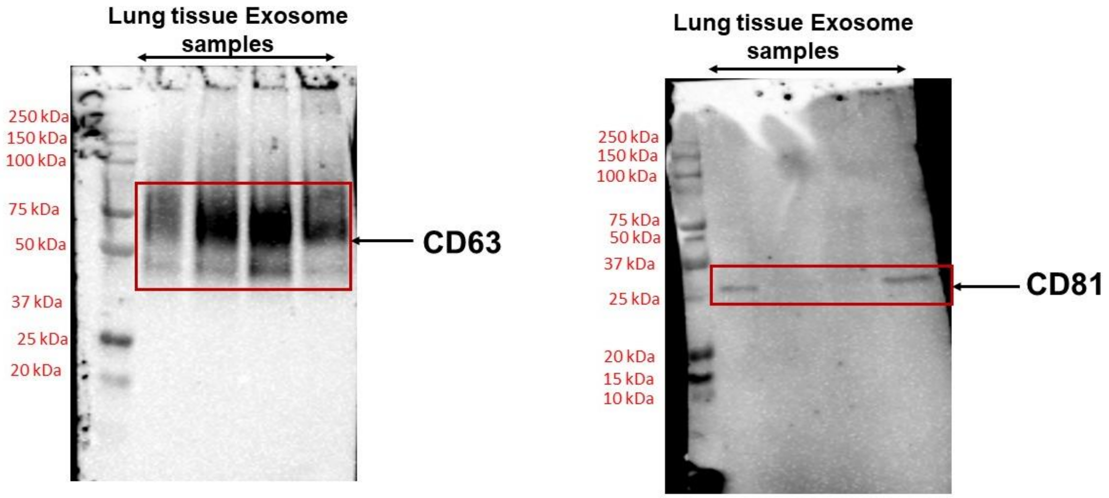

Supplementary Figure S2: Figure showing the original uncut blots for CD63 and CD81from lung tissue exosome fraction as shown in Figure 2(iii) of the manuscript.

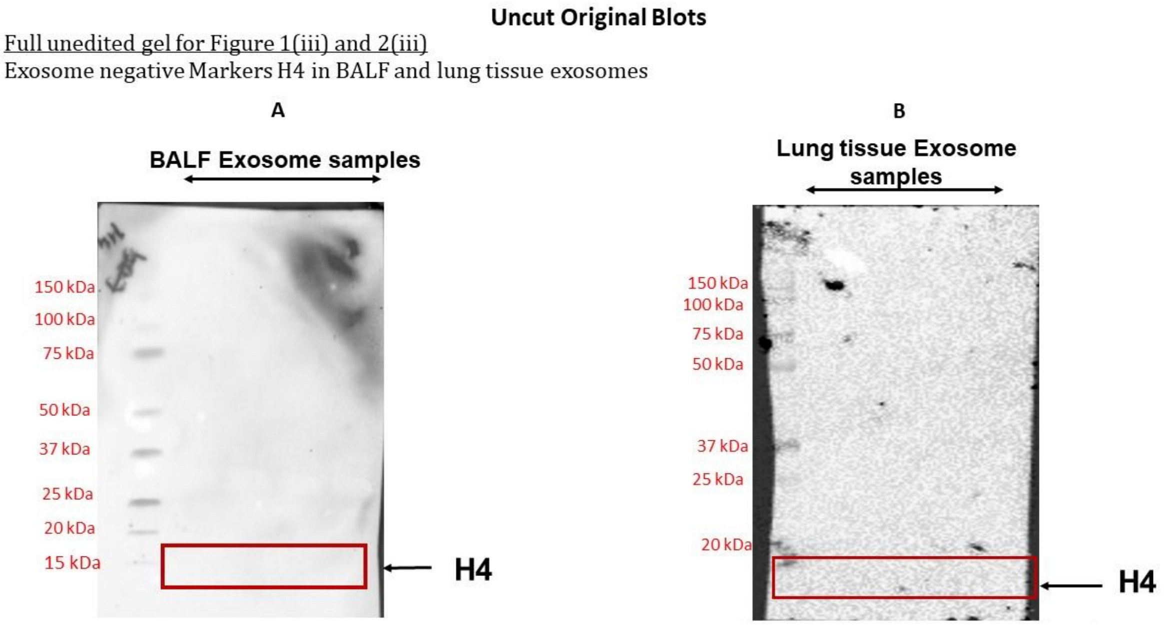

Supplementary Figure S3: Figure showing the original uncut blots for negative exosomal marker (H4) from (A) BALF and (B) lung tissue-derived exosome fraction as shown in Figures 1(iii) and 2(iii) respectively of the manuscript. NOTE: The blot probed for CD9 for BALF exosomes was stripped and re-probed for H4.

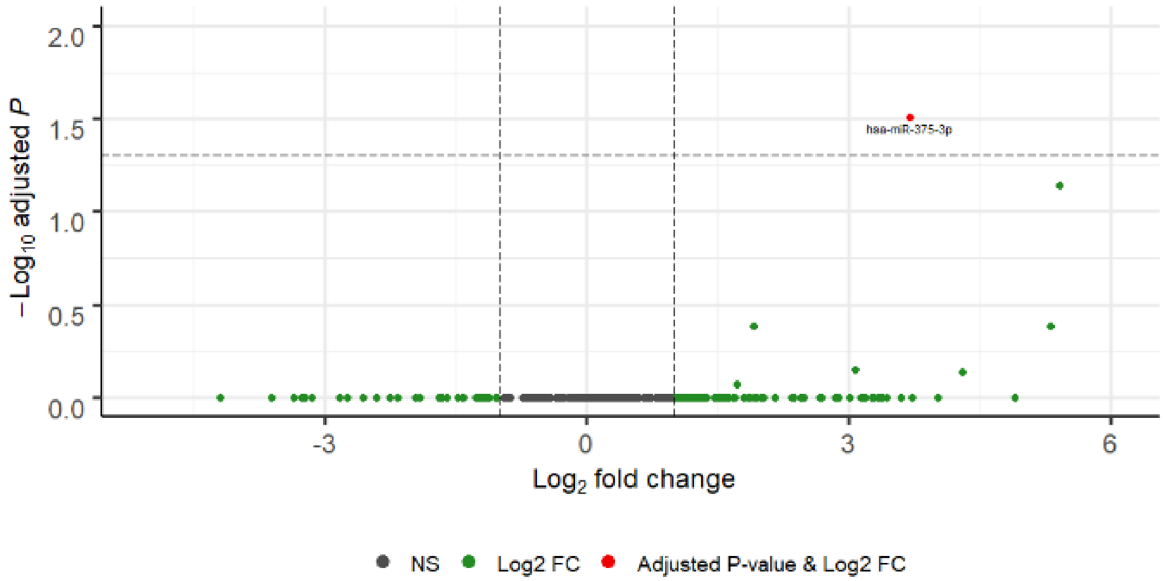

Supplementary Figure S4: Volcano plot showing differentially expressed miRNAs amongst BALF-derived exosomes from IPF vs COPD patients.

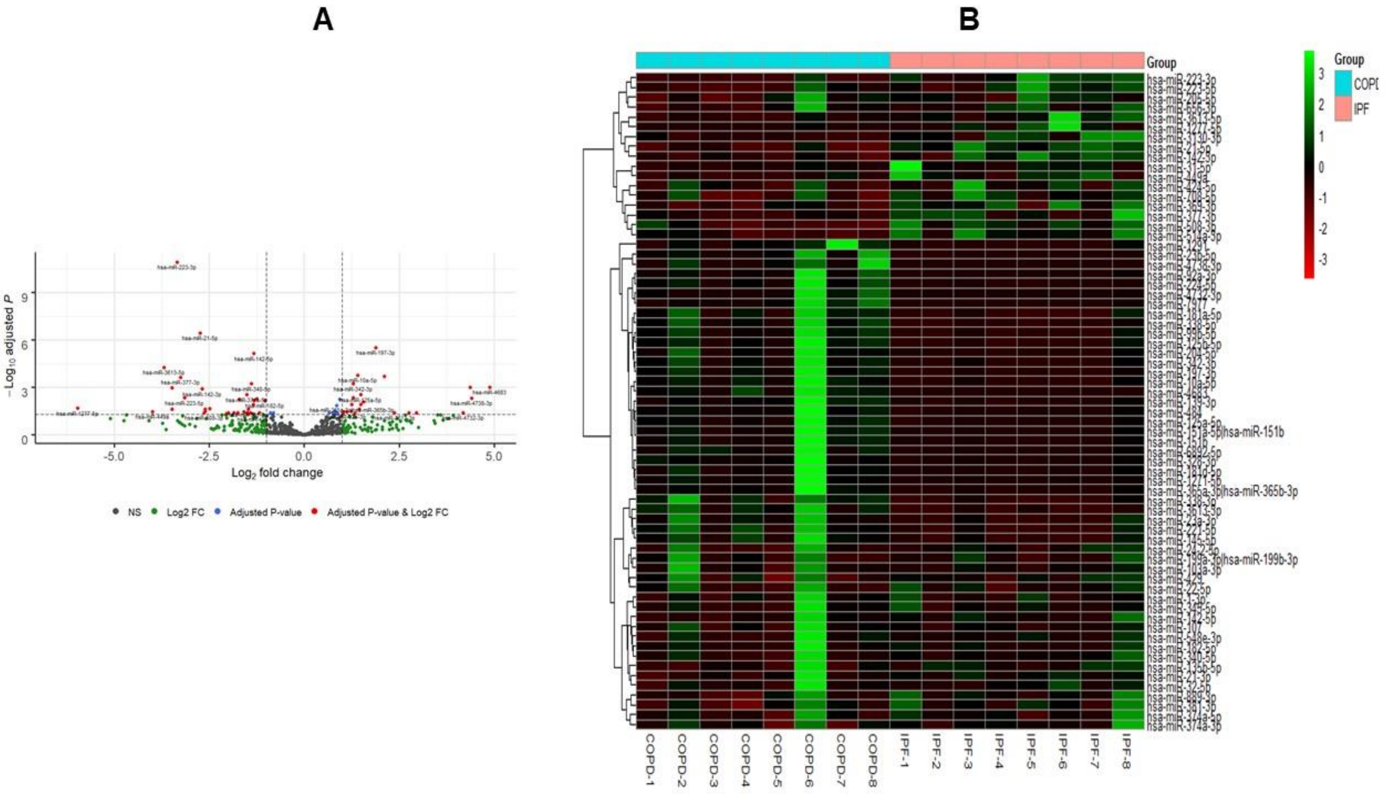

Supplementary Figure S5: (A) Volcano plot and (B) Heat map showing differentially expressed miRNAs amongst Lung tissue-derived exosomes from IPF vs COPD patients.
